# P20 COVID-19-associated pulmonary aspergillosis (CAPA): review of utility of local CAPA guideline and MDT in directing diagnosis and antifungal stewardship

**DOI:** 10.1093/jacamr/dlac004.019

**Published:** 2022-02-16

**Authors:** Lois Hawkins, Ting Yau, Tihana Bicanic, Marina Basarab

## Abstract

**Background:**

COVID-19-associated pulmonary aspergillosis (CAPA) is a recognized but incompletely characterized secondary fungal infection reported to be associated with increased mortality in patients in the ICU. Case definitions have been published but lack clarity: clinical and radiological features are non-specific and rely heavily on mycological tests for which both a timely availability and accuracy pose an issue contributing to the wide range of incidence reported of 4%–34%. In April 2021, we introduced a CAPA guideline for ICU patients at St George's University Hospital including when to suspect and how to investigate for CAPA (see Figure 1). The aim of this study was to evaluate its utility in identifying patients meeting ECMM_ISHAM criteria for possible/probable CAPA.

**Methods:**

Between March 2020 and May 2021 a retrospective audit was completed that identified all PCR-confirmed COVID-19 ICU patients prescribed either voriconazole, caspofungin, anidulafungin and AmBisome and reviewed their medical records, imaging and microbiology results categorizing patients into proven, probable, possible or unlikely CAPA based on ECMM/ISHAM criteria.

**Results:**

Twenty-one patients were prescribed the specified antifungals. All had CT imaging and varying microbiological investigations. Real-time clinical decisions on empirical antifungal prescribing for CAPA (start or stop) were made by a multidisciplinary team (MDT) comprising Microbiology and ICU physicians in light of clinical, microbiology and radiology findings. Nine of 21 patients prescribed empirical antifungals had radiological evidence of CAPA. *Aspergillus fumigatus* was cultured in six patients from bronchoalveolar lavage and two patients from sputum/trachea. Of these, seven were deemed significant and one attributed to colonization. Two patients were diagnosed with ‘probable’ and five as ‘possible’ CAPA, of whom one died. All seven patients received antifungal treatment for CAPA of varying duration (range 18–49 days). Only one patient had an underlying host risk factor (B cell lymphoma). Of those defined with probable/possible CAPA, average time from intubation to treatment was 18.4 days (range 8–31) and from onset of COVID symptoms to treatment was 28.5 days (range 18–49). Three of 21 patients were treated empirically for CAPA and all 3 died prior to further investigation and 15 patients following an MDT were felt to have unlikely CAPA. Serum BDG and GM were negative in all patients with probable/possible CAPA.

**Conclusions:**

The St George's CAPA guideline would have picked up two out of seven cases; two cases had antifungals started in another hospital so the utility of this guideline could not be assessed. Based on analysis of these cases the following changes were made to the guideline: (i) to assess patients ≥8 days post ICU admission (rather than ≥10 days) and (ii) assess patients following ≥3 days post appropriate antibiotic therapy (rather than following ≥5 days). Serum BDG and GM were negative in all patients with probable/possible CAPA prior to starting treatment therefore negative serum markers do not exclude diagnosis; this is consistent with experience reported elsewhere. Radiological presentations of CAPA were variable and wide ranging, and BAL sampling was not always possible, therefore we found the MDT approach including radiology, infection, respiratory and ICU invaluable in making decisions regarding treatment. This helped to reduce the use of fungal diagnostics, antifungal agents and appropriately manage patients with potential CAPA. The rationalization of antifungal use by the introduction of this guidelines was particularly important and also expedited the introduction of in-house fungal diagnostics.

**Figure 1.**
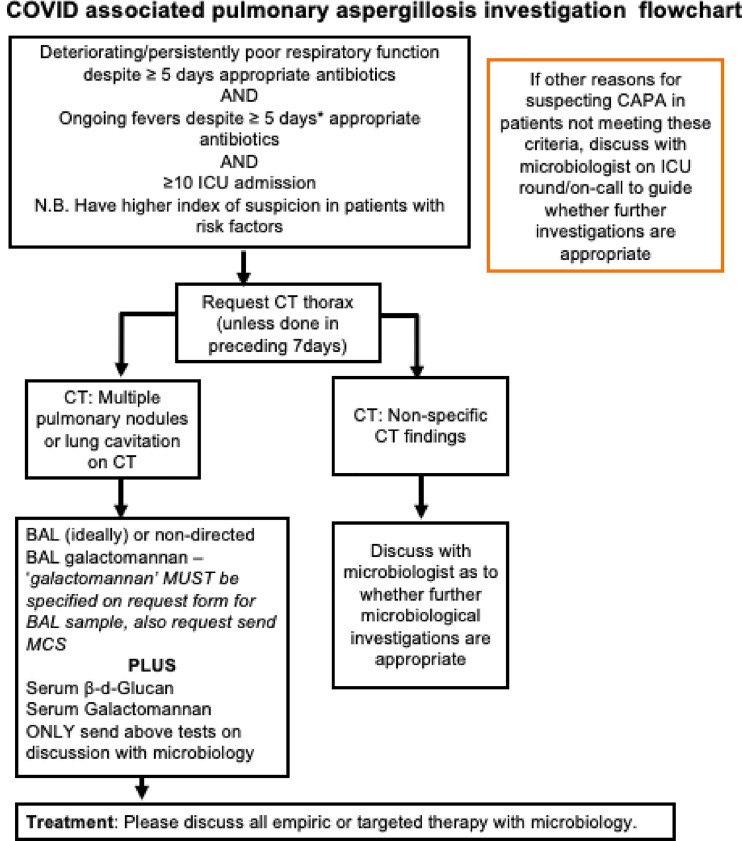
St George's Hospital CAPA Guideline 2021.

